# Cerebral Palsy: Early Markers of Clinical Phenotype and Functional Outcome

**DOI:** 10.3390/jcm8101616

**Published:** 2019-10-04

**Authors:** Christa Einspieler, Arend F. Bos, Magdalena Krieber-Tomantschger, Elsa Alvarado, Vanessa M. Barbosa, Natascia Bertoncelli, Marlette Burger, Olena Chorna, Sabrina Del Secco, Raye-Ann DeRegnier, Britta Hüning, Jooyeon Ko, Laura Lucaccioni, Tomoki Maeda, Viviana Marchi, Erika Martín, Catherine Morgan, Akmer Mutlu, Alice Nogolová, Jasmin Pansy, Colleen Peyton, Florian B. Pokorny, Lucia R. Prinsloo, Eileen Ricci, Lokesh Saini, Anna Scheuchenegger, Cinthia R. D. Silva, Marina Soloveichick, Alicia J. Spittle, Moreno Toldo, Fabiana Utsch, Jeanetta van Zyl, Carlos Viñals, Jun Wang, Hong Yang, Bilge N. Yardımcı-Lokmanoğlu, Giovanni Cioni, Fabrizio Ferrari, Andrea Guzzetta, Peter B. Marschik

**Affiliations:** 1 Research Unit iDN, Division of Phoniatrics, Medical University of Graz, 8036 Graz, Austria; magdalena.krieber@medunigraz.at (M.K.-T.); florian.pokorny@medunigraz.at (F.B.P.); peter.marschik@med.uni-goettingen.de (P.B.M.); 2 University of Groningen, University Medical Center Groningen, Beatrix Children’s Hospital, Division of Neonatology, 9713 GZ Groningen, The Netherlands; a.f.bos@umcg.nl; 3 National Rehabilitation Institute, Cerebral Palsy Department, 14389 Mexico City, Mexico; draelsaalvarado@gmail.com (E.A.); cvinals@inr.gob.mx (C.V.); 4 University of Illinois at Chicago, UI Health, Department of Occupational and Physical Therapy, Chicago, IL 60612, USA; vbarbo1@uic.edu; 5 University of Modena and Reggio Emilia, Department of Clinical and Surgical Sciences for Mothers, Children and Adults, Neonatal Intensive Care Unit, 41124 Modena, Italy; natascia.bertoncelli@gmail.com (N.B.); llucaccioni@unimore.it (L.L.); ferrarif@unimore.it (F.F.); 6 Stellenbosch University, Faculty of Medicine and Health Sciences, Department of Health and Rehabilitation Sciences, Cape Town 8000, South Africa; mbu@sun.ac.za; 7 IRCCS Fondazione Stella Maris, Department of Developmental Neuroscience, 56128 Pisa, Italy; ochorna@fsm.unipi.it (O.C.); sabrina.delsecco@gmail.com (S.D.S.); v.marchi@fsm.unipi.it (V.M.); gcioni@fsm.unipi.it (G.C.); a.guzzetta@fsm.unipi.it (A.G.); 8 Ann & Robert H. Lurie Children’s Hospital of Chicago, Chicago, IL 60611, USA; r-deregnier@northwestern.edu; 9 University Hospital Essen, Department of Pediatrics I, 45122 Essen, Germany; Britta.Huening@uk-essen.de; 10 Daegu Health College, Department of Physical Therapy, 41453 Daegu, Korea; julie0202@dhc.ac.kr; 11 Oita University Faculty of Medicine, Department of Pediatrics, 879-5593 Oita, Japan; tmaeda@oita-u.ac.jp; 12 Institute of Life Sciences, Scuola Superiore Sant’Anna, 56127 Pisa, Italy; 13 Children’s Rehabilitation Institute Teleton, 72825 Puebla, Mexico; martin@teleton-pue.org.mx; 14 The University of Sydney Medical School, Children’s Hospital at Westmead Clinical School, The Discipline of Child and Adolescent Health, Sydney 2050, NSW, Australia; CMorgan@cerebralpalsy.org.au; 15 The University of Sydney, Cerebral Palsy Alliance Research Institute, Sydney 2050, NSW, Australia; 16 Hacettepe University, Faculty of Physical Therapy and Rehabilitation, Developmental and Early Physiotherapy Unit, 06100 Ankara, Turkey; akmer@hacettepe.edu.tr (A.M.); bilgenuryardimci@hacettepe.edu.tr (B.N.Y.-L.); 17 Municipal Hospital of Ostrava, Children ’s Department, 72880 Ostrava, Czech Republic; alice.nogolova@mnof.cz; 18 Masaryk University, Faculty of Medicine, 62500 Brno, Czech Republic; 19 Medical University of Graz, Department of Pediatrics and Adolescent Medicine, Division of Neonatology, 8036 Graz, Austria; jasmin.pansy@medunigraz.at (J.P.); a.scheuchenegger@medunigraz.at (A.S.); 20 Northwestern University, Department of Physical Therapy and Human Movement Science, Chicago, IL 60611, USA; colleen.peyton1@northwestern.edu; 21 Cerebral Palsy Association Eastern Cape, Port Elizabeth 6001, South Africa; lurindap@cpaec.org.za; 22 University of New England/Maine LEND Program, Portland, ME 04103, USA; ericci@une.edu; 23 Post Graduate Institute of Medical Education and Research, Department of Pediatrics, Pediatric Neurology Division, Chandigarh 160012, India; drlokeshsaini@gmail.com; 24 Rede SARAH de Hospitais de Reabilitação, Reabilitação Infantil, 30510-000 Belo Horizonte, Brazil; cinthia.diniz.silva@gmail.com (C.R.D.S.); fabianautsch@gmail.com (F.U.); 25 Lady Davis Carmel Medical Center, NICU Developmental Follow-up Clinic, 34362 Haifa, Israel; marinaso@clalit.org.il; 26 University of Melbourne, School of Health Sciences, Department of Physiotherapy, Parkville 3052, Australia; aspittle@unimelb.edu.au; 27 Murdoch Children’s Research Institute, Parkville 3052, Victoria, Australia; 28 Kiran Society for Rehabilitation and Education of Children with Disabilities, Varanasi 221011, India; medico@kiranvillage.org; 29 Stellenbosch University, Faculty of Medicine and Health Sciences, Department of Paediatrics and Child Health, Cape Town 8000, South Africa; jivz@sun.ac.za; 30 Children’s Hospital of Fudan University, Department of Rehabilitation, Shanghai 201102, China; 13512183795@163.com (J.W.); hyang@shmu.edu.cn (H.Y.); 31 University Medical Center Göttingen, Child and Adolescent Psychiatry and Psychotherapy, 37075 Göttingen, Germany; 32 Karolinska Institutet, Department of Women’s and Children’s Health, Center of Neurodevelopmental Disorders (KIND), 11330 Stockholm, Sweden

**Keywords:** cerebral palsy, dyskinesia, fidgety movements, general movements, GMFCS, hemiplegia, hypotonia, identification, motor optimality score, segmental movements

## Abstract

The Prechtl General Movement Assessment (GMA) has become a cornerstone assessment in early identification of cerebral palsy (CP), particularly during the fidgety movement period at 3–5 months of age. Additionally, assessment of motor repertoire, such as antigravity movements and postural patterns, which form the Motor Optimality Score (MOS), may provide insight into an infant’s later motor function. This study aimed to identify early specific markers for ambulation, gross motor function (using the Gross Motor Function Classification System, GMFCS), topography (unilateral, bilateral), and type (spastic, dyskinetic, ataxic, and hypotonic) of CP in a large worldwide cohort of 468 infants. We found that 95% of children with CP did not have fidgety movements, with 100% having non-optimal MOS. GMFCS level was strongly correlated to MOS. An MOS > 14 was most likely associated with GMFCS outcomes I or II, whereas GMFCS outcomes IV or V were hardly ever associated with an MOS *>* 8. A number of different movement patterns were associated with more severe functional impairment (GMFCS III–V), including atypical arching and persistent cramped-synchronized movements. Asymmetrical segmental movements were strongly associated with unilateral CP. Circular arm movements were associated with dyskinetic CP. This study demonstrated that use of the MOS contributes to understanding later CP prognosis, including early markers for type and severity.

## 1. Introduction

Recent guidelines for the early identification of infants at risk for cerebral palsy (CP) recommend the Prechtl General Movement Assessment (GMA) in combination with neonatal magnetic resonance imaging (MRI) and the Hammersmith Infant Neurological Examination (HINE) as the assessments of choice [1]. Early identification aims at immediate referral for intervention to improve functional outcomes [2,3,4].

The Prechtl GMA [5] is a diagnostic tool based on visual Gestalt perception of videoed age-specific normal or abnormal general movements (GMs) with interscorer reliabilities ranging from Kappa 0.88 to 0.92 [5,6]. GMA can be applied from birth onward, but its high predictive power with sensitivity-values from 95% to 98% and specificity-values from 89% to 96% in cohorts at high risk for CP [7,8,9,10] primarily lies in the assessment of fidgety GMs. Fidgety GMs, or, in short, fidgety movements, are continuous small amplitude, moderate speed movements of shoulders, wrists, hips, and ankles in all directions and of variable accelerations in typically developing infants at 3–5 months post-term age [7,11]. Fidgety movements gradually disappear when voluntary, purposeful movements become predominant [5,12]. Abnormal fidgety movements, defined as having exaggerated speed, amplitude, and jerkiness, may point to neurological deficits, but their predictive power is low with a positive likelihood ratio LR + = 5.1 (95% CI: 1.5 to 17) [5,11]. It is the absence of fidgety movements that is strongly related to the development of severe neurological deficits (LR + > 51), most intensively studied as a predictor of CP [5,7,8,9,10,11]. With the focus on fidgety movements and their absence, the Prechtl GMA has become a cornerstone assessment for early identification of infants at risk for CP. 

The motor repertoire of infants at 3–5 months consists not only of fidgety movements but also of other movements such as antigravity movements to the midline and legs lift, kicking, swiping, or wiggling-oscillating limb movements [13]. A detailed assessment of these age-specific movements including fidgety movements and several postural patterns was proposed in 2004 [5] and has been applied to several high-risk groups (e.g., [6,14,15,16]) but also to typically developing infants [6,17,18]. The scoring is based on the optimality concept [19], with the advantage that its semi-quantitative approach documents small changes in an otherwise categorically assessed motor behavior. Some of the studies applying this approach revealed that a reduced score was associated with motor and language dysfunction at toddler age [6,15], minor neurological dysfunctions [20], or learning difficulties at school age [21]. Two other studies [22,23] showed that such a detailed assessment of early movements and postures helps to predict the level of self-mobility in children with CP. In a total of 102 children, a non-optimal motor behavior was indicative of poor independent mobility five to 12 years later. Combining these studies [22,23], the number of children with bilateral spastic CP was reasonably high (*n* = 87), but only 14 children with unilateral CP and one child with dyskinesia were described. Apart from the lack of fidgety movements, infants who had poor independent mobility later were characterized by an atypical age-specific motor repertoire [22]. These infants showed repetitive opening and closing of the mouth [23], atypical finger postures [23], monotonous kicking [22], and a cramped-synchronized movement character [16,22,23]. The latter refers to stiff, monotonous movements of almost simultaneous contraction followed by almost simultaneous relaxation of at least two limbs and the trunk [5]. Infants with an increased risk of dyskinesia showed circular arm movements often with finger spreading and no fidgety movements [24]. Infants who went on to develop unilateral CP showed an asymmetry of segmental movements, which were reduced in the wrist and fingers contralateral to the lesion [25,26,27]. Such an early topographical differentiation including the motor type is clinically of utmost importance as early intervention strategies can be specifically tailored to the individual needs [1]. However, these promising results were based on rather small samples, i.e., 12 infants later diagnosed with dyskinesia [24], 16 preterm infants with unilateral intra-parenchymal echodensity, of whom 13 developed unilateral CP [25], and two different cohorts comprising 24 infants born at term with neonatal arterial ischemic cerebral infarction, of whom 14 developed unilateral CP [26,27]. As early identification of specific markers is crucial for early referral to intervention services, our first aim was to see if the results found in small samples can be replicated in a larger sample. Apart from providing specific early support for families, most parents or caregivers will want to learn about the severity of their child’s future physical disability. 

Second, a few authors [28,29,30] proposed that the high predictive values of the GMA might be only achievable within the expert group closely linked to the GM Trust. In order to reach a generalizability of our findings, we therefore paid special attention to include several study sites outside the centers where GM Trust experts are involved. Our endeavor was to contact colleagues who have been applying GMA for several years and are also certified for detailed GMA. We sent out an open call to centers that apply GMA in a standard manner to contribute to this study by providing convenience samples of individuals who prospectively underwent GMA and have now a reliable diagnosis of CP. 

Our primary objective was to associate movement and postural patterns assessed at 3–5 months in a worldwide collected sample of individuals later diagnosed with CP, with their functional ability and activity limitation as classified on the Gross Motor Function Classification System (GMFCS) ([31]; expanded and revised [32]). Our secondary objective was to confirm, in a large series of individuals—assessed worldwide by various local certified scorers—previous described predictors for bilateral spastic CP [22,23,33], unilateral spastic CP [25,26,27], and dyskinesia [24]. Specific questions were whether apart from absent fidgety movements (a) a cramped-synchronized movement character lasting until 3– 5 months predicts non-ambulant CP, (b) the results of previous studies [22,23] in smaller samples can be replicated, (c) an asymmetry of segmental movements indicate specifically unilateral CP, and (d) whether early markers for dyskinesia are different from those for later spasticity—in particular, whether circular arm movements and spread fingers are specific for later dyskinesia. Furthermore, based on an appropriate sub-sample size, this study aimed to find out whether (e) it is possible to determine specific motor or postural patterns indicative of ataxia or hypotonia. 

## 2. Methods

### 2.1. Study Design

We conducted an observational study on a convenience sample of 468 children who received a diagnosis of CP between 2012 and 2019. All individuals participated in follow-up and/or research programs in the various centers and had received a GMA at 9–22 weeks post term age (Median 13, IQR 12–15). For the purpose of this study, their video recordings have been re-assessed by one to five GM Trust certified scorers (advanced level including detailed GMA) at the respective study site. The scorers were not aware of the individuals’ functional mobility and activity limitation classified on the GMFCS-E&R at the time of scoring. The study followed the Strengthening the Reporting of Observational Studies in Epidemiology (STROBE) reporting guideline. 

### 2.2. Setting

In order to replicate previous findings that were obtained in significantly smaller samples [22,23,24,25,26,27], the first author sent a call for participation to 29 worldwide scattered sites where certified scorers (advanced level including detailed GMA) routinely use GMA. Twenty-seven sites (93%) responded, one site declined participation due to lack of capacity, and two potentially participating sites had to be excluded due to the lack of suitable data (i.e., children were too young for GMFCS-E&R assessment). Hence, the study was conducted in 24 study sites (83%), i.e., (a) six sites in Europe at the Medical University of Graz, Austria (including cases from the Graz University Audiovisual Research Database for the Interdisciplinary Analysis of Neurodevelopment, GUARDIAN, which currently comprises more than 2000 standardized GMA data sets collected worldwide); the University of Groningen, the Netherlands; the University Hospital Essen, Germany; the Municipal Hospital Ostrava, Czech Republic; the University of Modena and Reggio Emilia in Modena, Italy; the IRCCS Fondazione Stella Maris and the University of Pisa, Italy; (b) six study sites in North America at the University of New England/Maine in Portland, ME; the Ann & Robert H. Lurie Children’s Hospital of Chicago; the University of Chicago, Comer Children’s Hospital; and the University of Illinois in Chicago, IL, USA; the National Rehabilitation Institute in Mexico City; and the Children’s Rehabilitation Institute Teleton in Puebla, Mexico; (c) one study site in South America at the SARAH Network of Rehabilitation Hospitals in Belo Horizonte, Brazil; (d) two study sites in Africa at the Stellenbosch University in Cape Town; and the Cerebral Palsy Association Eastern Cape in Port Elizabeth, South Africa; (e) seven study sites in Asia at the Lady Davis Carmel Medical Center in Haifa, Israel; the Hacettepe University in Ankara, Turkey; the Post Graduate Institute of Medical Education and Research in Chandigarh, India; the Kiran Society for Rehabilitation and Education of Children with Disabilities in Varanasi, India; the Daegu Health College, South Korea; the Children’s Hospital of Fudan University in Shanghai, China; the Oita University, Japan; and (f) two study sites in Australia at the Murdoch Children’s Research Institute and University of Melbourne; and the University of Sydney Medical School and the Cerebral Palsy Alliance in Sydney. 

### 2.3. Participants

**Figure 1:**
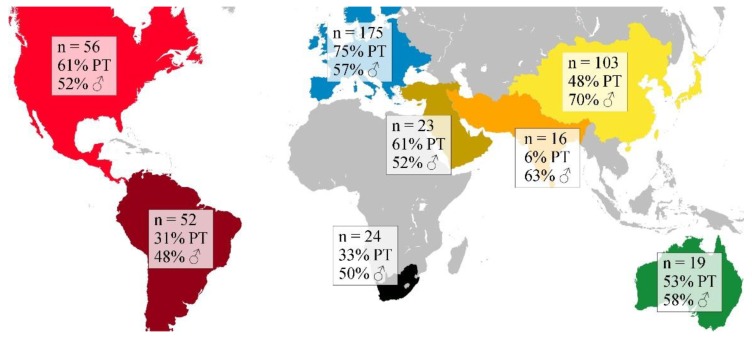
Regions of origin of participants (absolute numbers per region), including percentage (%) of individuals born preterm (PT) and percentage (%) of males (♂). For Asia, we provide information on three of the five geographical subregions: Western Asia (sand color), South Asia (orange), and East Asia (yellow).

The study comprised a total of 468 individuals (56% born preterm; 58% male) whose GMs were prospectively videoed and analyzed between 2011 and 2017. None of them participated in any of the previous studies on early signs of CP [16,22,23,24,25,26,27,33]. Gestational age ranged from 23 to 42 completed weeks (Median 34, IQR 29-39). Data on birthweight were available for 390 infants and ranged from 440 g to 4500 g (Median 2150, IQR 1198-3100). The majority of the participants (*n* = 463; 98.9%) were from high-risk groups comprising preterm birth and/or neonatal encephalopathy (white matter injury, cortical and deep grey matter lesions, stroke), signs of perinatal asphyxia such as delayed cry after birth (in low- and middle-income countries), hypoglycemia, jaundice, sepsis and neonatal infections (meningitis, pneumonia), neonatal seizures, a family history of CP, multiple birth, assisted reproduction, maternal and/or fetal virus infection, prenatal exposure to maternal diabetes, maternal substance abuse, and low socioeconomic status. The remaining five infants were referred to GMA from concerned parents (*n* = 2) or professionals (*n* = 3). Information on congenital birth defects is available for 224/468 participants (47.9%); 14/224 (6.3%) had a congenital birth defect (eight infants had a congenital brain malformation; four infants had a cardiac malformation, and two infants had congenital talipes equinovarus). Information on prenatal exposure to maternal drugs is available for 176/468 participants (37.6%). Four mothers had a psychosis and were treated with antipsychotic medication during pregnancy. Two infants were prenatally exposed to methamphetamines and/or opiates, and one infant to maternal chemotherapy. Twenty of 225 infants (8.9%) were twins, and three infants (1.3%) were one of triplets. For the remaining 243/468 participants (51.9%), no information on multiple gestation is available. Infants with chromosomal abnormalities were excluded. The regions of origin of all participants are given in Figure 1. 

Diagnoses of CP comprised (a) spasticity (*n* = 415; 88.7%; 59% born preterm, 59% male); (b) dyskinesia (*n* = 26; 5.5%; 31% born preterm; 42% male) including dystonia (*n* = 6); (c) mixed type (spasticity and dyskinesia) without definition of the predominant motor type (*n* = 20; 4.3%; 35% born preterm; 55% male); (d) ataxia (*n* = 2; 0.4%; both born preterm; one male); and (e) hypotonia (*n* = 5; 1.1%; two born preterm; two males). Dyskinesia, ataxia, and hypotonia affect all four limbs, whereas spasticity was categorized topographically as (i) unilateral (hemiplegia; *n* = 92/415; 22.2%; 64% born preterm; 50% male) and (ii) bilateral, including diplegia (lower limbs were more affected than upper limbs; *n* = 104/415; 25.1%; 75% born preterm; 67% male), and quadriplegia (all four limbs and trunk affected; *n* = 219/415; 52.7%; 49% born preterm; 59% male). Cognitive, speech-language, or other developmental impairments and co-morbidities were not taken into consideration. Activity limitation as classified on the GMFCS-E&R was distributed as follows: level I (*n* = 107; 22.9%; 66% born preterm; 54% male); level II (*n* = 55; 11.8%; 69% born preterm; 62% male); level III (*n* = 60; 12.8%; 72% born preterm; 63% male); level IV (*n* = 86; 18.4%; 58% born preterm; 56% male); and level V (*n* = 160; 34.1%; 38% born preterm; 58% male). Given that we collected a global convenience sample (Figure 1) with incomparable neonatal intensive care management approaches and other confounders such as prenatal care or maternal nutrition, we refrained from analyzing group differences in terms of gestational age.

### 2.4. Variables

Individuals were categorized according to their GMFCS-E&R level but also grouped into independent ambulation (GMFCS levels I to II; *n* = 162; 35%) and mobility dependent on a device (walking device or wheelchair; GMFCS levels III to V; *n* = 306; 65%). To test the secondary aims, we further categorized individuals into unilateral (*n* = 92; 20%) vs. bilateral CP (*n* = 376; 80%) and into spastic (*n* = 415; 93%) vs. non-spastic CP (*n* = 33; 7%). For the latter comparison, we did not include the mixed type group (*n* = 20) as it was not possible to determine their primary and secondary motor type. In order to understand specific markers for children with dyskinetic CP, i.e., research question (d), we matched each child with dyskinesia (*n* = 26) to two children with spasticity (*n* = 52) according to their GMFCS level, age at GMA recording, preterm or full-term delivery, and gender. 

### 2.5. General Movements Assessment, GMA

**Figure 2:**
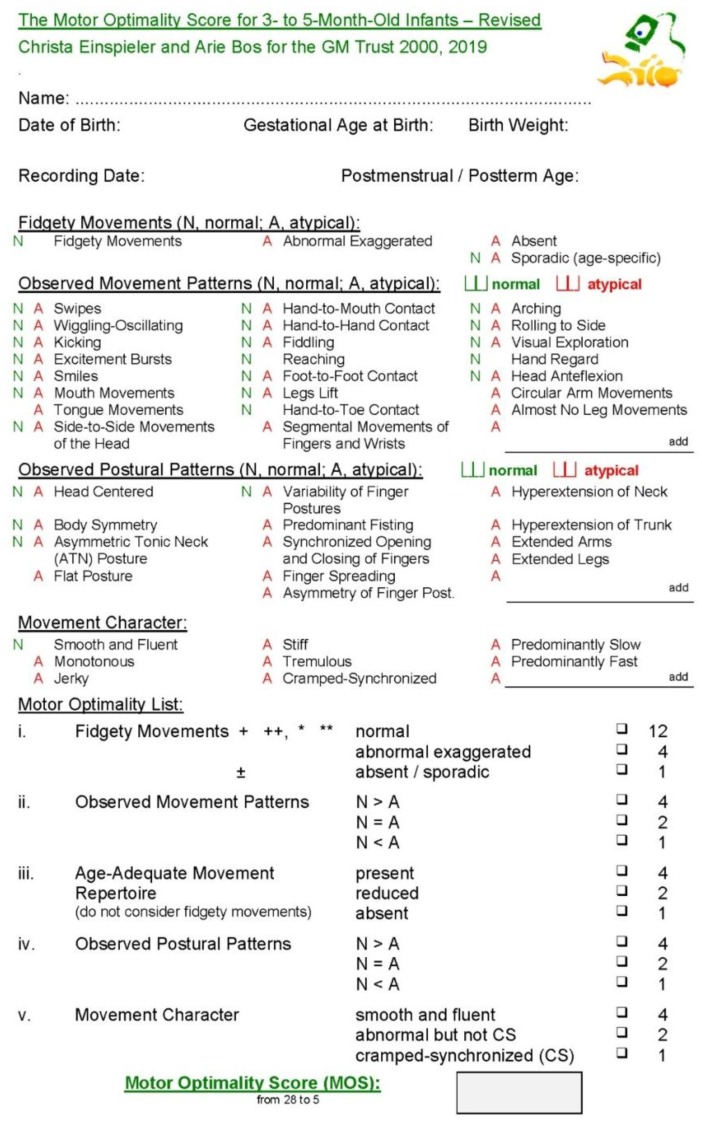
Consensus revised version of the Assessment of the Motor Repertoire at 3–5 months in order to obtain the Motor Optimality Score.

At 9–22 weeks post term age (Median 13, IQR 12-15), infants were videotaped (at the respective study site or using remote GMApp technology [34,35] at the participants’ home) for 2–5 min of active wakefulness (not crying, not fussy, not sucking on a pacifier), lying in supine position, and without interaction [5]. The detailed GMA for three- to five-month-old infants ([5], p.26 and revised version as Figure 2) comprises the following five subcategories: (i) temporal organization and quality of fidgety movements, scored as normal (score 12), abnormal exaggerated (score 4), or absent (score 1); (ii) quality of movement patterns other than fidgety movements (Table 1, above) scored as predominantly normal (score 4), equal number of normal and atypical patterns (score 2), or predominantly atypical (score 1); (iii) age-adequate movement repertoire, scored as present (score 4), reduced (score 2), or absent (score 1); criteria are given in Table 2; (iv) postural patterns (Table 1, below) scored as predominantly normal (score 4), equal number of normal and atypical patterns (score 2), or predominantly atypical (score 1); and (v) movement character scored as smooth and fluent (score 4), monotonous and/or jerky, stiff, tremulous, slow/fast (score 2), or cramped-synchronized (score 1). Adding the scores of the five subcategories reveals the Motor Optimality Score (MOS) with a maximum of 28 (i.e., best possible performance) and a minimum of 5. An MOS from 25 to 28 is considered to be optimal; scores < 25 are considered to be reduced [6,17,36]. The intra-observer reliability is high with intra-class correlation coefficients ranging from 0.80 to 0.98 [15,37] and Cohen Kappa ranging from 0.87 to 0.91 [20].

### 2.6. Functional Assessment of the Neurological Findings 

At a median age of three years and five months (IQR two years and 10 months–five years and seven months), all individuals were age-specifically scored and reliably classified by neurologists, rehabilitation doctors or developmental pediatricians using the Five-Level Gross Motor Function Classification System—Expanded & Revised (GMFCS-E&R; www.canchild.ca). The GMFCS-E&R describes the movement ability of a child with CP in one of five levels across five age ranges. It documents the child’s functional ability and performance in different settings, particularly during sitting, walking, and wheeled mobility. The lower the level, the more independent the child’s gross motor abilities [31,32]. 

### 2.7. Ethics

The study was approved by the institutional review boards of all participating centers (cf. Subchapter 2.2). Parental written informed consent for recording, storage, and scientific use of video data was obtained for all individuals at the point of GMA. The accompanying medical history data (gender, preterm birth, motor type and topography of CP, GMFCS-E&R level) were abstracted from medical records. 

### 2.8. Statistical Analysis

First, we evaluated descriptive parameters including medians and interquartile ranges (IQR) or percentages to summarize sample characteristics. The MOS (and its subcategories) and the GMFCS-E&R are ordinal data. Number of normal or atypical movements or postural patterns, gestational age at birth, recording, and assessment age are interval data. All other GMA data (movement and postural patterns) and outcome data (activity limitation in two categories, topography, motor form) were analyzed as categorical variables. 

Comparisons between categorical variables were made using Pearson Chi-square test or Fisher’s exact test (two sided). Odds ratios (OR) and 95% confidence intervals (CI) were calculated for motor patterns that significantly differentiated outcome variables. To determine whether two or more independent samples had the same distribution in MOS and/or GMFCS-E&R, we applied the Mann Whitney U test or Kruskal Wallis test. To determine whether the quantities of normal and atypical movements and postures (dependent samples; not normally distributed) were equally distributed, we used the Wilcoxon signed-rank test. 

The association between the MOS and the GMFCS-E&R levels was examined using Spearman’s rank-order correlations. To test the predictive power of the MOS-subcategories (polytomous ordinal predictors) on the GMFCS outcomes (polytomous ordinal criterion), we ran ordinal regression analysis. The predictive power of abnormal movement and postural patterns (metric predictors) on GMFCS outcomes (polytomous ordinal criterion) was again estimated by means of ordinal regression analysis.

Statistical significance at *p* < 0.05 was assumed throughout. Data analyses were performed using IBM SPSS Statistics 25 (SPSS Inc., Chicago, IL, USA).

### 2.9. Data Availability

The datasets generated and analyzed during the current study are available from the corresponding author on reasonable request.

## 3. Results

### 3.1. The Motor Optimality Score and Its Association with GMFCS Outcomes 

The MOS ranged from five to 24 with a Median = 7 (IQR 6-9). None of the infants scored in the optimum range, but 14 infants had a score of 20 and higher (3%), and nine of these children were eventually diagnosed with unilateral CP. Eleven of 14 infants with an MOS ≥ 20 score were classified as GMFCS level I, and the remaining three as level II. 

Spearman’s rank-order correlation indicates a strong association between overall MOS and GMFCS-outcome of *rho* = −0.66 (*p* < 0.001). This holds equally true for male and female participants. The association is stronger for infants born at term (*rho* = −0.68; *p* < 0.001) than for infants born preterm (*rho* = 0.60; *p* < 0.001). 

**Figure 4:**
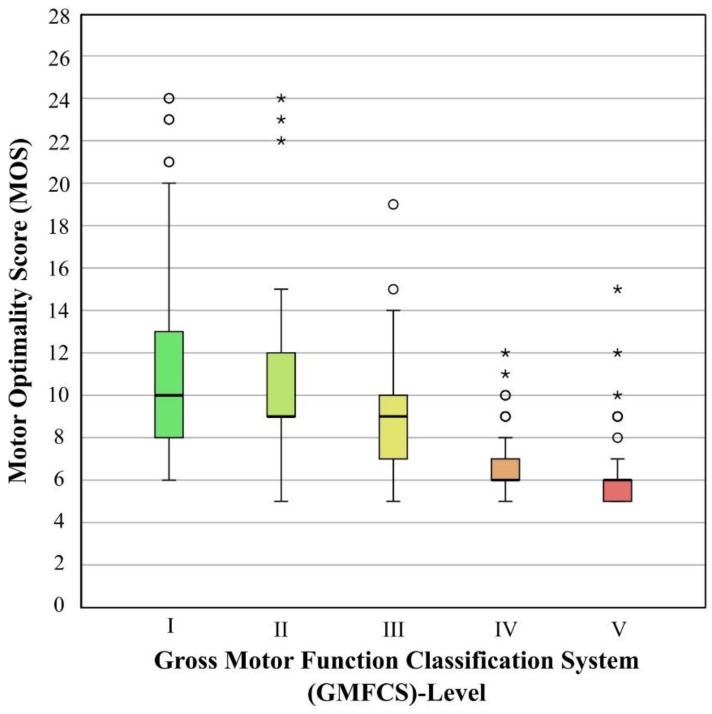
Distribution of the Motor Optimality Score (MOS) within the levels of the Gross Motor Function Classification System—Expanded & Revised (GMFCS-E&R). Whiskers indicate an interquartile range (IQR) × 1.5. Key: ° outliers (within 3 × IQR), * extreme outliers (>3 × IQR).

As the majority of infants (95%) did not have fidgety movements, we also analyzed the association between the “MOS minus subcategory fidgety movements” (with a maximum score 16) and the GMFCS outcomes. This revealed a similar result of *rho* = −0.64 (*p* < 0.001). As suggested by quartiles, an MOS > 14 was most likely associated with GMFCS outcomes I or II, whereas GMFCS outcomes IV or V were hardly ever associated with an MOS > 8 (Figure 4).

The combined predictive power of the MOS-subcategories on GMFCS outcomes was estimated by means of an ordered logit model. The model fitting information suggests a significant improvement of the final model compared to the intercept-only model (Chi-square = 236.02; *p* < 0.001), and the test of parallel lines indicates the model to be in line with the proportional odds assumption (Chi-square = 21.81; *p* = 0.113). Pseudo R-square parameters (Nagelkerke = 0.416; Cox and Snell = 0.396) suggest the MOS-subcategories to explain a substantial amount of variance in GMFCS outcomes. With parameter estimates ranging between −0.19 and −1.26, increases in the scores for (i) fidgety movements, (ii) quality of movement patterns, (iv) postural patterns and (v) movement character significantly decrease the likelihood of higher GMFCS outcomes (Table 3). That is, higher scores in these predictors are associated with higher gross motor functioning. The increase in odds of changing from a lower to a higher GMFCS level for each one-unit increase in the rating of the predictors was lowest for (v) movement character (0.29), followed by (ii) quality of movement patterns (0.50), (iv) postural patterns (0.68) and (i) fidgety movements (0.83). The (iii) age-adequacy of the movement repertoire, however, may not significantly contribute to explain variation in GMFCS outcomes when the other four predictors are included in the model (Table 3).

### 3.2. Fidgety Movements and Outcome 

Eighteen infants (3.8%) had normal fidgety movements, and five infants (1.1%) showed abnormal exaggerated fidgety movements. The other 445 infants (95.1%) did not display fidgety movements. The age of GMA recording did not impact our results (Kruskal Wallis test; *p* = 0.506). 

Eleven infants with fidgety movements developed unilateral spastic CP, five infants developed diplegia, one infant developed spasticity in all four limbs, and one infant was later diagnosed with hypotonia. Their GMFCS-E&R levels were I in 14 children, II in three children, and III in the child with spasticity in all four limbs. The latter was born to a diabetic mother at term with hypoxic-ischemic encephalopathy (Sarnat grade II); the infant started to have infantile spasms at 6 months of age and was diagnosed with West syndrome. 

All five children with abnormal exaggerated fidgety movements were later diagnosed with spasticity: one with unilateral CP, level I; one with diplegia, level IV; and three with quadriplegia, level III.

The quantity of atypical movement patterns did not vary between infants with or without fidgety movements (Kurskal Wallis test; *p* = 0.286). Infants with normal fidgety movements had more other normal movement patterns (Median 5, IQR 3-6) than infants with abnormal fidgety movements (Median 4, IQR 1-6) and infants without fidgety movements (Median 1, IQR 0-3; Kruskal Wallis test; *p* < 0.001). Also, postural patterns were more often normal in infants with normal fidgety movements (Median 2, IQR 2-3) than in infants with abnormal exaggerated fidgety movements (Median 2, IQR 2-2) or no fidgety movements at all (Median 1, IQR 1-2, Kruskal Wallis test; *p* < 0.05). The quantity of atypical postural patterns was not associated with the presence of fidgety movements (Kruskal Wallis test; *p* = 0.065). 

### 3.3. Quality of Movement Patterns and Age-Adequate Repertoire 

The occurrence of normal movement patterns was rare in this sample of infants with CP (Median 1, IQR 0-3). Only one infant exhibited ten normal movement patterns and was later diagnosed with unilateral CP, GMFCS level I. Atypical movement patterns (Median 2, IQR 1-3) occurred more often than normal movement patterns (Median 1, IQR 0-3; Wilcoxon signed-rank test, *p* < 0.001). Spearman’s rank-order correlation indicates a strong negative association between the quantity of normal movement patterns and GMFCS outcomes (*rho* = −0.574; *p* < 0.001), and a moderate positive association between atypical movement patterns and the GMFCS outcomes (*rho* = 0.300; *p* < 0.001). The quantity of normal (*p* = 0.638) and atypical movement patterns (*p* = 0.184) did not differ between infants later diagnosed with spastic vs. non-spastic CPs (Mann Whitney U test). Infants who were later diagnosed with unilateral CP had more normal movement patterns (Median 3, IQR 2-5) than infants later diagnosed with bilateral CP (Median 1, IQR 0-3; Mann Whitney U test, *p* < 0.001). The quantity of atypical movement patterns was not related to the topography of CP (*p* = 0.185). The contribution of each movement pattern and its OR is given in Table 4. Here, we summarize only those movement patterns which revealed at least a two-fold increased risk at the lower level of the 95% CI: (a) atypical mouth movements were associated with GMFCS outcomes III–V and bilateral CP; (b) atypical foot-to-foot contact was associated with GMFCS outcomes III–V and bilateral CP; (c) atypical arching was associated with GMFCS outcomes III–V and bilateral CP ; (d) atypical visual exploration was associated with GMFCS outcomes III–V; (e) normal hand regard was associated with a later diagnosis of unilateral CP; (f) an asymmetry of segmental movements was associated with GMFCS outcomes I–II and unilateral CP; and (g) circular arm movements were associated with GMFCS outcomes III–V, bilateral CP, and non-spastic CP (Table 4). 

Only 24 infants (5.5%) had an age-adequate movement repertoire (for age-specific criteria see Table 2); 43 infants (9.2%) had a score 2 for the repertoire, and the remaining 401 infants (85.7%) did not show enough age-specific movement patterns and revealed a score 1. For the quality of movement patterns, we observed a different picture. Although the number of normal movement patterns was low (Median 1, IQR 0-3), there were still 140 infants (29.9%) with a predominance of normal movement patterns; 73 infants (15.6%) had an equal number of normal and atypical movement patterns; but more than the half of the infants (*n* = 255; 54.5%) showed predominantly atypical movement patterns. 

### 3.4. Postural Patterns 

Whereas the number of normal postural patterns was low (Median 1, IQR 1-2) the number of atypical postural patterns ranged from 0 to 10 (Median 4, IQR 3–5; Wilcoxon signed-rank test, *p* < 0.001). Spearman’s rank-order correlation indicated an association between both quantity of normal (*rho* = −0.384; *p* < 0.001) and quantity of atypical postural patterns and the GMFCS-outcome (*rho* = 0.415; *p* < 0.001). Compared to infants with a later diagnosis of bilateral CP (Median 1, IQR 1–2), infants who developed unilateral CP had more normal postural patterns (Median 2, IQR 1–3; Mann Whitney U test *p* < 0.001). The opposite held true for atypical postures: infants later diagnosed with unilateral CP had fewer atypical postures than infants later diagnosed with bilateral CP (Median 3, IQR 2–4 vs. Median 4, IQR 3–5; Mann Whitney U test, *p* < 0.001). Whereas the quantity of atypical postural patterns did not differ between children later diagnosed with spastic or non-spastic CP (*p* = 0.190), infants later diagnosed with non-spastic CP had fewer normal postural patterns than infants later diagnosed with spastic CP (Median 1, IQR 0–2 vs. Median 2, IQR 1–3; Mann Whitney U test, *p <* 0.01). The contribution of each postural pattern and its OR is provided in Table 5. Again, we summarize only those postural patterns which revealed at least a two-fold increased risk at the lower level of the 95% CI: (a) atypical head posture was associated with GMFCS outcomes III–V; and (b) the lack of variable finger postures was associated with GMFCS outcomes III–V (Table 5). 

### 3.5. The Movement Character

One infant was scored to move smoothly and fluently. His outcome was diplegia, GMFCS level I. A cramped-synchronized movement character was strongly associated with GMFCS outcomes III–V and bilateral CP (Table 6). Infants with cramped-synchronized movements had also more often atypical kicking (*p* < 0.001), atypical arching (*p* < 0.05), atypical legs lift (*p* < 0.001), atypical trunk rotation (*p* < 0.001), and atypical head anteflexion (*p* < 0.001). None of the infants with a cramped-synchronized movement character had an asymmetry of segmental movements (*p* < 0.001). The contribution of the other items describing the movement character is given in Table 6. 

### 3.6. Early Markers of Unilateral CP

The activity limitation as classified on the GMFCS-E&R for the 92 children with unilateral CP was as follows: level I (*n* = 64; 69.6%), level II (*n* = 19; 20.7%), level III (*n* = 4; 4.3%), and level IV (*n* = 5; 5.4%).

As hypothesized, asymmetrical segmental movements were strongly associated with unilateral CP (Table 4). Fifty-eight infants (63%) who later developed unilateral CP had a higher frequency of segmental movements in the ipsilesional (unaffected) arm. Within the group of children with unilateral CP, the asymmetry of segmental movements was not related to the GMFCS outcome (Pearson Chi-square; *p* = 0.624). The remaining 34 infants (37%) had an almost equal amount of segmental movements on their lesional and non-lesional side. 

As 11/92 (12%) infants had normal fidgety movements, we further assessed if the admittedly small group of infants with fidgety movements and a later diagnosis of unilateral CP differed from those who did not show fidgety movements with respect to any motor pattern. No differences were found. An asymmetry of segmental movements indicating the impaired side (less segmental movements) was observed in five of eleven infants who had fidgety movements (*p* = 0.209). 

### 3.7. Are Early Signs for Dyskinesia Different From Those for Spasticity? 

The activity limitation as classified on the GMFCS-E&R for the 26 children with dyskinesia was as follows: level I (*n* = 1; 3.8%), level II (*n* = 1; 3.8%), level III (*n* = 5; 19.2%), level IV (*n* = 11; 42.3%), and level V (*n* = 8; 30.9%). 

In order to distinguish movement patterns among children with and without spastic CP, we selected the group of children with dyskinetic CP and created a 1:2 comparison group of children with spastic CP matched for GMFCS outcomes. Further matching criteria were age at GMA, preterm or full-term delivery, and gender. None of the infants later diagnosed with dyskinesia and none of the comparison group had developed fidgety movements. Apart from circular arm movements, none of the other movement patterns discriminated between both groups. Circular arm movements occurred in 15/26 (58%) infants later diagnosed with dyskinesia but only in 8/52 (15%) matched infants who later developed spastic CP (*p* < 0.001). Considering the total sample of children with spastic (*n* = 415) and non-spastic CP (*n* = 33), the OR for circular arm movements and non-spastic CP was 8.77 (Table 4). 

From the postural patterns, the item “body symmetry” revealed a significant result: 88% of infants with a later diagnosis of dyskinesia had an atypical body posture compared to 60% of infants with a later diagnosis of spasticity (*p <* 0.01). Also, the ATN posture occurred more often in infants with a later diagnosis of dyskinesia (42% vs. 17%, *p <* 0.05). Finger spreading did not distinguish between the two groups (*p* = 0.818). Postural patterns were more often atypical in infants with a later diagnosis of dyskinesia (Median 4, IQR 3-5 vs. Median 3, IQR 3-5; Mann Whitney U test; *p* < 0.05). 

With only one exception, no infant later diagnosed with dyskinesia, was assessed as jerky during 3–5 months post-term age, whereas a jerky character was assigned to 23% of the matched comparison group (*p* < 0.05). 

The MOS did not differ between these two groups. Both groups had a Median 7 (IQR 6–9; Mann Whitney U test; *p* = 0.702). 

### 3.8. Does the Early Motor Repertoire Reveal Markers for Ataxia or Hypotonia? 

The sample is far too small to answer this question. The two children diagnosed with ataxia were assigned to GMFCS level IV and V. Both had no fidgety movements, and their MOS was 5 and 10, respectively. The infant with the MOS = 5 had a cramped-synchronized movement character, which so far is mainly described as a predictor for spastic CP [1,5,7,33]. 

The GMFCS levels for the five children with hypotonia were as follows: level II (*n* = 1), level IV (*n* = 2), and level V (*n* = 2). As already mentioned in Section 3.2, one infant later diagnosed with hypotonia showed fidgety movements (his outcome was GMFCS level II); the other four did not. Consequently, the MOS showed a big variation ranging from 6 to 22. The infant with fidgety movements was born at term with hypoxic ischaemic encephalopathy; MRI revealed mild basal ganglia injury. 

Interestingly, none of the five infants later diagnosed with hypotonia showed antigravity movements of the upper extremity (such as hand-to-mouth contact, hand-to-hand contact, fiddling). However, foot-to-foot contact and/or legs lift occurred in three infants. A flat posture was scored in only one infant. 

## 4. Discussion

### 4.1. Lower Optimality Scores Justify Referral to Treatment Programs 

The results of our worldwide multicenter study confirm that GMA is advisably considered the reference standard for early identification of a high risk for CP. Infants with an MOS ≤ 14 who do not develop fidgety movements should be confidently referred to targeted early treatment programs during the period of greatest neuroplastic changes [3,4,40,41]. Importantly, infants with marked asymmetry in fidgety expression and segmental movements, indicative of probably unilateral CP, should also be referred for early intervention as they are responsive to treatment in this early phase [42,43,44]. 

That an MOS < 8 is associated with GMFCS levels IV and V is in agreement with previous results obtained in much smaller samples [16,22,23]. Although normative data on the MOS are not yet available, we do have information from several studies who had applied the MOS in a total number of 563 neurotypical controls [6,14,17,18,36] that the median MOS of typically developing infants ranged from 26 to 28 (with IQRs ranging from 21–28 to 28). Hence, the median MOS = 7 (IQR 6–9) of our sample is clearly reduced, and it is obvious that the absence of fidgety movements had significantly contributed to such a low score. However, the *absence* of a movement pattern by itself cannot predict the degree of later functional limitations. That the correlation between MOS and GMFCS outcomes (*rho* = −0.66) was similar to the correlation that we obtained when we had not taken fidgety movements into consideration (*rho* = −0.64) reflects the role of the concurrent motor behavior. It is the combination of several atypical movement and postural patterns with an often monotonous or even cramped-synchronized movement character and the lack of fidgety movements that contributes to various degrees of a reduced MOS being predictive for various GMFCS outcomes. 

### 4.2. Absent Fidgety Movements, a Reliable Predictor for Cerebral Palsy

Since its first introduction more than 30 years ago [7], the presence or absence of fidgety movements remains an early marker for normal or adverse neurodevelopment (especially CP) [1,8,22,23,24,25,26,27,33,41,45,46]. The first studies considered mainly infants with a later diagnosis of spasticity. However, after Einspieler et al. [24] reported that also infants with a later diagnosis of dyskinesia had not developed fidgety movements, it was clear that intact cortico-spinal fibers and the output from the basal ganglia are necessary to generate normal fidgety movements. Although very small in numbers, we now can add that cerebellar lesions also impact fidgety movements. MRI studies demonstrated that brain lesions associated with absent fidgety movements were manifold: white matter abnormalities [25,47,48], reduced cerebellar transverse diameter [49], arterial infarctions in the territory of the middle cerebral artery [26,27], but also basal ganglia and thalami damage associated with white matter changes with or without cortical injury [33] have been described in infants born very preterm or in infants after perinatal asphyxia who did not show fidgety movements. As a result, we are still far away from understanding the specific neural mechanisms responsible for the generation of fidgety movements. 

As Kwong et al. [45] very recently described a few infants whose fidgety movements were scored absent at 12 weeks but present and normal at 14 weeks, it is important to note here that the age of GMA recording did not impact our results. Although abnormal exaggerated fidgety movements are often associated with later coordination difficulties, fine manipulative disabilities, as well as autism spectrum disorder [11], they have also been reported in a small number of infants who were later diagnosed with CP [7,11]. Hence, we hypothesized a small number of infants showing abnormal fidgety movements, which was indeed around 1%. 

A frequently asked question is whether infants with normal fidgety movements can also develop CP. As the sensitivity-values for CP range from 95% to 98% [7,8,9,41], the answer is yes. Mild, usually unilateral CP (GMFCS I or II) were exceptionally reported in infants who had shown normal fidgety movements [7,8,23,27,41,50,51]. Burger and co-workers [46] described one infant with normal fidgety movements who developed spastic diplegia, GMFCS I. Our report is the first on the possibility that—although rare—an infant with normal fidgety movements can also develop quadriplegia (GMFCS level III; it is important to note that this child was diagnosed with West syndrome at six months of age) or hypotonia (GMFCS Level II). As a rule, however, normal fidgety movements along with a smooth concurrent motor performance indicate a neurotypical development [5,11]. 

### 4.3. Movement and Postural Patterns: Quantity Counts but Quality is More Relevant

Brain lesions may alter the cortical modulation exerted on the regions responsible for generating fidgety movements and other spontaneous movement patterns. That might explain why few normal movements and/or postural patterns and an exceedingly high number of atypical movement and/or postural patterns were associated with GMFCS outcomes III to V, indicating poorer functional ability. Vice versa, the more normal and the less atypical the movement and/or postural patterns, the better the gross motor function outcome of the child. Similar findings have been reported previously for 15 infants later diagnosed with CP [20]. The quantity of normal and atypical patterns differentiated between CP and a neurotypical outcome as well as the diagnosis of minor neurological dysfunction in school children who were born preterm [20].

Some movement patterns such as reaching or hand-to-toe contact were never observed, others occurred in a very small number of infants and most often atypically (Table 4). Among them were anteflexion of the head (observed in 2.5%), hand regard (5%), excitement bursts (8%), arching (9%), rolling to the side (10%), or hand-to-hand contact (10%). These behaviors tend to require either whole body or visuomotor coordination, which may be why they were infrequently or atypically displayed in our sample of infants later diagnosed with CP. Only smiles and hand-to-mouth contact occurred more often normally than atypically. 

The most frequently occurring movement patterns (again comprising normal and atypical performance) were visual exploration (54%), mouth movements (49%), kicking (48%), and side-to-side movements of the head (47%). Visual exploration was scored atypical if strabismus, nystagmus, setting sun phenomenon or other abnormal eye movements were observed; such atypical eye movements had an OR = 4.32 for GMFCS outcomes III to V (Table 4). A previous study that applied the GMA in a high-risk group of preterm infants [52] reported that strabismus occurred in half of all infants with a later diagnosis of CP; the high odds found in our study for co-occurrence of atypical eye movements and GMFCS III–V is, however, new. Another recent study [53] noted that strabismus was described to occur more frequently in children with a diagnosis of CP, levels IV or V, than in children with GMFCS levels I–III. That atypical mouth movements were strongly associated with GMFCS outcomes III–V (OR = 6.50) confirmed previous findings [23] and are reflective of a more extensive brain injury. In fact, almost one third of our cohort showed repetitive opening and closing of the mouth and atypical tongue movements (OR = 1.65) such as repetitive licking or long-lasting tongue protrusion (Table 4). It is obvious that coordinated and variable oral movements are not only crucial for successful consumption of food and liquid but also for the production of sounds and speech. Consequently, early recognition of atypical mouth and tongue movements may indicate a need for early intervention to improve oral motor functions. 

Although atypical kicking occurred in more than one third of our cohort (Table 4), the OR for GMFCS outcomes III–V was only 2.60. Also, Bruggink et al. [22] reported monotonous repetitive kicking associated with GMFCS outcomes III–V. Furthermore, infants with extensive lesions in the periventricular and lobar white matter showed a decreased variability on spatial and temporal parameters of kicks [54]; they were unable to dissociate tight couplings among the hip, knee, and ankle, unlike typically developing children [55]. In our revised definition of atypical kicking (Figure 2; Table 1), we now include the presence of dissociated or coupled kicking in the detailed scoring, which may be an early indicator of selective motor control. As many children in our sample with CP were likely to have damage in the periventricular and/or white matter, we would expect most to have difficulty dissociating couplings of the hip, knee, and ankle. 

While arching has previously been associated as an early clinical sign of CP [56], in our population of 468 infants with CP, the presence of arching was rare (8.2%; Table 4). However, when atypical arching was observed (7.1%, Table 4), the OR for GMFCS outcomes III–V was 26.00. Prolonged and overly stiff arching was also related to a cramped-synchronized movement character; the latter might also explain atypical kicking or rolling to side *en bloc*.

Bruggink et al. [22] reported that a score 1 for the MOS-subcategory (iii) age-adequacy of the repertoire (indicating the age-adequacy is absent) was associated with GMFCS outcomes IV and V. Our study, however, demonstrated that this subcategory didn’t significantly contribute to explain the variation in GMFCS outcomes (Table 3). The reason for these diverging results could be different scoring approaches. Whereas Bruggink et al. scored the age-adequacy of the repertoire as “1” when fewer than five normal movement and postural patterns were observed [22], we followed the age-related approach given in Table 2. As a result, our findings may be reflective of the age-related motor deficits seen across all GMFCS levels in the first two years of life. 

Bruggink et al. [22] also discussed a flat posture with rare antigravity movements of the limbs being associated with GMFCS outcomes I and II, and a persistent ATN posture related to GMFCS outcomes III–V. In our study, the odds for ATN and outcomes were slightly increased but the flat posture did not affect outcomes (Table 5). Interestingly, the presence of persistent ATN posture has been frequently attributed to an early sign of CP in infancy [56], but 75% of infants with CP did not have a persistent ATN (Table 5). Over 30 years ago, Harris [57] noted that difficulty in maintaining the head in the midline appeared to be an early sign for CP. Indeed, we found an OR = 3.29 for atypical head posture to be associated with GMFCS outcomes III–V. 

Fisting is a predominant hand posture in newborns and young infants [58], but its presence failed to discriminate between low-risk and neurological abnormal infants [39]. In our cohort, fisting occurred predominantly, i.e., more than 80% of the observation time, in 37.4% of all children with CP (Table 5) but was not associated with their functional ability. A lack of variability in finger movements and postures, however, was associated with GMFCS outcomes III to V (OR = 5.96, Table 5), and has been also discussed in the context of abnormal visual-spatial perception and poorer cognition [18,21]. Also, Konishi and Prechtl [39] reported that complex finger movements resulting in variable finger postures occurred more often in low-risk preterm infants than in neurologically abnormal infants. Variable finger movements and postures are related to the maturation and function of the corticospinal tract [39]. Guzzetta et al. [27] suggested that an impoverishment of complex and variable finger movements might be an early functional consequence of an impaired development of the corticospinal projections to the spinal cord. Another atypicality concerning finger movements and postures is finger spreading, which had been described to discriminate between later spasticity and dyskinesia [24]. In our sample, spread fingers occurred in only 18.6% with an OR = 2.76 for GMFCS outcomes III–V (Table 5) but were not related to non-spastic CP. 

Considering the movement character, cramped-synchronized movements have by far the highest OR for GMFSC-outcomes III to V (11.69) and bilateral CP (OR = 27.39). Over 15 years ago, Ferrari et al. [59] reported a 100% sensitivity and 92.5% to 100% specificity for cramped-synchronized movements, which usually start to occur around moderate to late preterm age and may last until 3–5 months or even longer [5,33]. One of Ferrari’s important contributions to GMA was that an early appearance and long duration of cramped-synchronized movements were associated with GMFCS outcomes IV and V [59]. Subsequent studies confirmed these findings [22,23,33]. Another study applying the MOS in 41 high-risk infants admitted to a rehabilitation clinic in Japan yielded MOS values between 5 and 7 in those five children who were later diagnosed with CP; all five children were scored as cramped-synchronized at their three- to five-month assessment, and four of them had GMFCS level V at later assessment [16]. Most probably, infants with a cramped-synchronized movement character (18.8% of our cohort) were also scored as monotonous and stiff because multiple answers for subcategory (v) were possible. Nevertheless, a stiff movement character occurred in more than half of all infants and monotonousness in 82.3% (Table 6). Although we found a high rate of occurrence in our population of children with CP, a monotonous movement character should not be considered a specific marker for CP. Other studies have found monotonous movements at 3–5 months, which occurred in children with non-CP diagnoses including infants later diagnosed with autism [60], or lower working memory capacity and lower processing speed [61], in infants prenatally exposed to maternal medication [62], or infants with genetic disorders [63,64]. 

### 4.4. An Asymmetry of Segmental Movements Indicate a High Risk for Unilateral Cerebral Palsy 

Cioni and colleagues [25] were the first to describe a reduction of segmental movements on the side contralateral to the lesion in 15/16 preterm born infants with a unilateral intraparenchymal echodensity. A few years later, Guzzetta et al. [26,27] confirmed Cioni’s results in two small samples of full-term infants with neonatal cerebral infarction who eventually developed unilateral CP. Such early asymmetries might be due to the regression of the transient ipsilateral corticospinal projections [65]. At the same age, fine distal movements might come under the control of corticospinal fibers, which originate from the contralesional frontal and parietal cortex and descend laterally to the spinal cord, resulting in small and elegant wrist and finger movements [25]. 

Until now, we have paid special attention to the asymmetry of segmental movements in infants who did not show fidgety movements [5]. The present study yielded 18 children (3.8%) who developed CP although they had developed fidgety movements. Actually, 11 of them were later diagnosed with unilateral CP, and five children had asymmetrical segmental movements early indicating the side of their functional impairment. Although small in numbers and statistically not significant, in the future, we must continue to explore an asymmetry of segmental movements in spite of the presence of normal fidgety movements. Such an asymmetry can be clinically relevant even if brain imaging does not indicate unilateral or indeed any other form of CP [8,66] in order to guarantee allocation to early targeted intervention for improving manual abilities [1,3,4,40,42,43,44]. As a word of caution, we must also be aware that 34 infants with a later diagnosis of unilateral CP (37%) did not show an asymmetry of segmental movements and among them were six infants who had developed fidgety movements. Hence, some cases of unilateral CP may be missed by GMA. 

### 4.5. Circular Arm Movements and an Atypical Body Posture are Associated with Dyskinesia 

Circular arm movements have an OR = 8.77 for non-spastic CP. In fact, more than half of all infants (58%) with a later diagnosis of dyskinesia had circular arm movements. By contrast to a first study on early markers of dyskinesia [24], finger spreading did not co-occur with circular arm movements and did not differentiate between later dyskinesia and spasticity. 

It is important to realize that the remaining 42% of infants with a later diagnosis of dyskinesia did not display circular arm movements. This is a much higher proportion than the 25% documented in the previous study [24]. Furthermore, no other movement pattern discriminated between them and infants later diagnosed with spasticity. Children with later dyskinesia had, however, more atypical postural patterns during early infancy than their peers with a later diagnosis of spasticity. It was especially an asymmetric body posture, which occurred more frequently in infants with a later diagnosis of dyskinesia. To date, there has not been a published case with an outcome of dyskinetic CP who had shown fidgety movements. In accordance with previous documentations [7,23,24,50], all of the children with dyskinesia in this study, including the two children with GMFCS outcomes I or II, were also scored with absent fidgety movements. 

### 4.6. Limitations 

The strength of our study lies in the detail with which we examined movements and postures at 3–5 months in a large, worldwide collected sample of infants later diagnosed with CP. As we are dealing with a global convenience sample there are, however, some limitations. First, access to prenatal care and neonatal intensive care managements are not comparable. Hence, as also mentioned in 2.3., we refrained from analyzing group differences in terms of preterm vs. full-term birth. We could also not focus on influencing environmental factors such as socio-economic status, teratogens, drug intake, viral infections, or malnutrition, to name but a few potential confounders. Some of these factors have been investigated with respect to GMA [46,67], but their impact on the MOS is yet to be studied. Second, this being a worldwide study, one might consider the participants’ varied ethnic backgrounds as problematic. However, we are dealing with spontaneous, endogenously generated movements. It is therefore very unlikely that different care-giving practices had an impact on the early motor patterns in question. Furthermore, fidgety movements have been assessed worldwide for more than 20 years with similar cross-cultural results [6,7,16,23,36,41,46,67]. Third, although the number of individuals with spasticity in our sample (89%) mirrors the usual rate of occurrence (85–91% [1]), individuals with dyskinesia (10% in our sample) are slightly overrepresented (vs. 4–7% [1]), whereas individuals with ataxia (usually 4–6% [1]) and hypotonia (usually 2% [1]), who are not routinely diagnosed in all countries [1], are clearly underrepresented in our sample. We are therefore not able to describe early markers for ataxia and hypotonia. Forth, during the re-assessment of the GM videos, local settings were heterogeneous. For instance, in two sites, only one certified scorer was available, while some other sites included up to five different raters. A few disagreements (*n* = 5) were discussed with the first author and finally solved. As these situations mirror routine GMA use, eventual fluctuations in scoring accuracies between the study sites might be negligible. 

## 5. Conclusions and Future Aspects

A low MOS (including the absence of fidgety movements) in an infant with a high-grade brain lesion predicts the development of CP and even a severe activity limitation but does not yet, in itself, allow a definitive diagnosis. According to recent guidelines of early identification and early intervention in CP [1], GMA should be combined with MRI. This is, however, not available at all times and in all places. When neuroimaging is not available, observation comes into play: absent fidgety movements, an MOS ≤ 14, a cramped-synchronized movement character, and/or the asymmetry of segmental movements of fingers and wrists certainly indicate a need for early intervention and supporting families in a timely manner. At all times, however, we need to accept that perfect prediction of outcomes cannot exist, as the brain is highly plastic during early infancy, and there are many factors that influence a child’s overall motor outcome, including nutrition, environment enrichment, parental involvement, early intervention, and other medical treatments. 

As families’ differing socio-economic backgrounds are considered to affect the severity of CP [68,69], especially in low income settings [70], we plan to enlarge our worldwide cohort with the aim to find out if different social, economic, and cultural situations such as caregiving procedures alter the early motor repertoire and hence might impact the association between early movements and postures and the severity of CP. 

## Figures and Tables

**Figure 3:**
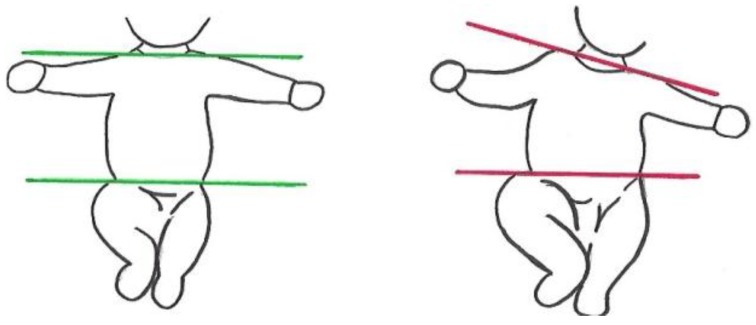
Illustration of the postural item “Body Symmetry”. Score normal if an imaginary line through the shoulder joints and an imaginary line through the hip joints run parallel (left; green lines). Score atypical if this is not the case (right, red lines).

**Table 1:** Definition of movement and postural patterns to be scored at 3–5 months [5,13,22,23,24,25,26,27,38,39].

Movement Pattern	Definition
Swipes	Ballistic-like movements with a sudden onset but fluid course and smooth offset; can go in downward or upward direction; most noticeable in extended arms; but also in partially or fully extended legs; large amplitude and high speed.
	Score atypical if repetitively occurring in more than one third of the observation time.
Wiggling-Oscillating Movements	Oscillatory, uniplanar movements; most noticeable in partially or fully extended arms but from time to time also in partially extended legs, with a frequency of 2–3 Hz; small amplitude and moderate speed.
	Score atypical if repetitively occurring in more than one third of the observation time.
Kicking	Can occur in a single leg and/or as simultaneous bilateral kicking with a fast flexion phase followed by a slower extension phase with decoupling of hip, knee and ankle.
	Score atypical if monotonous and/or coupling of hip, knee, and ankle is observed such that all joints tend to flex or extend in temporal synchrony.
Excitement Bursts	Wiggling-oscillating movements and/or swipes co-occur with kicking and are accompanied by a pleasurable and excited facial expression.
	Score atypical if monotonous and without pleasure mimic.
Smiles	Score atypical if awkward and frozen.
Mouth Movements	Are variable and usually related to vocalization (cooing) and/or non-nutritive sucking.
	Score atypical if opening and closing occur repetitively.
Tongue Movements	Score atypical if tongue protrusion is repetitive and/or long-lasting.
Side-to-Side Movements of the Head	The head crosses the midline smoothly and fluently. Do not mark if the head moves only from midline to side and back. Score atypical if repetitive.
Hand-to-Mouth Contact	The arm is moved against gravity and the hand touches the mouth with or without finger inserted. Do not mark if the head is on the side and the arm is not moved against gravity. Score atypical if repetitive.
Hand-to-Hand Contact	Both hands are brought together in the midline and the fingers of both hands repetitively touch, stroke or grasp each other. Score atypical if asymmetrical, or if both hands are fisted.
Fiddling	The fingers of one or both hands repetitively touch, stroke or grasp an object, most often the own clothing. Score atypical if the infant touches an object or the own clothing, no finger movements occur, and the hand has difficulties to release.
Reaching	One or both arms intentionally extend to some object in the immediate environment; the fingers may or may not contact the surface of the object. (This behavior is not elicited by a tester but occurs spontaneously.)
Foot-to-Foot Contact	Legs are elevated and feet are brought together with plantar-to-plantar touching from time to time. Do not mark if the feet remain on the surface during contact. Score atypical if foot-to-foot contact occurs mainly on the tibial side and/or is characterized by repetitive rubbing.
Legs Lift	Both legs lift vertically upward; partial or full extension at the knees; hips and pelvis are slightly tilted upward; one or both hands may touch or grasp the knees; sometimes it occurs together with anteflexion of the head. Score atypical if it occurs stiff and without variation.
Hand-to-Toe Contact	One or both hands touch, stroke or grasp the toes.
Segmental Movements of Fingers and Wrists	Independent movements of fingers and/or movements of moderate speed at the level of the wrist joint including rotation, palmar flexion and extension, and ulnar or radial flexion. Score atypical if asymmetrical.
Arching	After the soles touch the surface, the infant extends the back and neck causing a full trunk and head curve to form. Sometimes locomotion occurs. Do not mark if arching is a sign of discomfort. Score atypical if arching is prolonged and/or too stiff.
Rolling to Side	As a result of the soles of the feet pushing down on the lying surface, one side of the hip is lifted and rotated. From about 18 weeks onwards, the whole body is turned from supine to prone lying in a movement started by the head. Sometimes the infant returns to supine lying. Score atypical if the infant moves head and pelvis simultaneously sideways upward, moves the top leg forward and topples over *en bloc*, and/or if rolling is non-intentionally.
Visual Exploration	The infant visually explores the environment. Score atypical, if abnormal eye movements, (transient) strabismus, nystagmus, and/or setting sun phenomenon occur. Each atypical eye movement pattern is given a separate atypical mark; for instance, if the child has both nystagmus and setting sun phenomenon, give two atypical scores.
Hand Regard	The infant visually attends to the movements of his/her hand(s).
Head Anteflexion	The head is moved against gravity, sometimes the chin touches the trunk. Score atypical if prolonged and too stiff.
Circular Arm Movements	Uni- or bilateral, monotonous, slow forward rotations of the semi-flexed or extended arms, starting in the shoulder. They occur with or without spread fingers.
Postural Pattern	Definition
Head Centered	The head can be kept centered for at least 10 s; chin and sternum are in one line. Score atypical if the head cannot be centered, i.e., is tilted or in lateral position.
Body Symmetry	An imaginary line through the shoulder joints and an imaginary line through the hip joints run parallel (Figure 3). Score atypical if this is not the case throughout the recording.
Asymmetric Tonic Neck (ATN) Posture	The ATN posture cannot be observed or the extended arm can be easily flexed without turning the head. Score atypical if each spontaneous side movement of the head elicits an ATN that cannot be overcome by flexion of the extended arm.
Flat Posture	Lying in supine, all four limbs are mainly on the surface; antigravity movements and flexion in hips and knees are rare; arms and legs hardly move above the level of the trunk.
Variability of Finger Postures	Postures of the fingers, which result from isolated movements of one finger, simultaneous movements of two or three fingers, and/or sequential movements of two or more fingers. Fisting might occur from time to time. Score atypical if finger postures are rare and/or lack variability.
Predominant Fisting	Score atypical if fisting occurs more than 80% of the observation time. In this case also variability of finger postures is scored “atypical”.
Synchronized Opening and Closing of Fingers	Bilateral simultaneous extension of all fingers away from the palm is followed by bilateral flexion of all fingers towards the palm.
Finger Spreading	Unilateral or bilateral abduction and extension of all fingers.
Asymmetry of Finger Postures	Score atypical if one hand shows different finger postures and the other hand is fisted.
Hyperextension of Neck and/or Trunk	Do not mark if the infant focusses his/her attention to an object or a person in the right or left upper corner.
Extended Arms	Bilateral predominant extension of the arms on or above the surface.
Extended Legs	Bilateral predominant extension of the legs on or above the surface.

**Table 2:** How to score the age-adequate movement repertoire, i.e., subcategory (iii) of the Motor Optimality Score, in three- to five-month-old infants.

	9 to 11 Weeks PTA	12 to 13 Weeks PTA	14 to 15 Weeks PTA	16 Weeks PTA and Older ^a^
Score 4	at least four normal movement patterns	at least four normal movement patterns including normal foot-to-foot contact	at least four normal movement patterns including normal foot-to-foot contact and normal hand-to hand contact	at least four normal movement patterns including the following three obligatory patterns: normal foot-to-foot contact and normal hand-to-hand contact and normal legs lift
Score 2	three normal movement patterns	at least four normal movement patterns but not foot-to-foot contact	at least four normal movement patterns including normal foot-to-foot contact or normal hand-to hand contact	at least four normal movement patterns including only two of the above-mentioned obligatory movement patterns
Score 1	less than three normal movement patterns	less than four normal movement patterns	normal foot-to-foot contact and normal hand-to-hand contact are not observable	only one of the above-mentioned obligatory movement patterns is present or all of them are absent

Normal mouth movements are not taken into consideration. Key: PTA, post term age in completed weeks; ^a^ In case the infant rolls intentionally from supine to side or prone, foot-to-foot contact and/or legs lift does not need to be present anymore; for a score 4 rolling to side and hand-to-hand contact plus at least two other normal movement patterns are required.

**Table 3:** Parameter estimates for the ordered logit model. Estimated coefficients, standard errors (SE), Wald statistics, *p*-values, and 95% confidence intervals (CI) for the five motor optimality score (MOS) subcategories.

Subcategories of the MOS	Estimated Coefficient	SE	Wald Statistics	*p*-Value	95% CI
(i) Fidgety Movements	−0.19	0.06	11.43	0.001	−0.30 to −0.08
(ii) Quality of Movement Patterns	−0.68	0.08	71.71	<0.001	−0.84 to −0.52
(iii) Age-Adequate Movement Repertoire	−0.16	0.15	1.16	0.282	−0.44 to −0.13
(iv) Postural Patterns	−0.36	0.08	19.20	<0.001	−0.52 to −0.20
(v) Movement Character	−1.26	0.25	24.75	<0.001	−1.76 to −0.76

**Table 4:** Movement patterns at 3 to 5 months and their normal vs. atypical occurrence in 468 infants later diagnosed with cerebral palsy (CP).

Movement Pattern	Normal Quality *n* (%)	Atypical *n* (%)	Not Observed *n* (%)	GMFCS-E&R Levels I–II (*n* = 162; 34.6%) vs. III–V (*n* = 306; 65.4%)	Unilateral (*n* = 92; 19.7%) vs. Bilateral (*n* = 376; 80.3%) CP	Spastic (*n* = 415; 92.6%) vs. Non-Spastic (*n* = 33; 7.4%) CP ^a^
Swipes	57 (12.2%)	34 (7.3%)	377 (80.5%)	OR_GMFCS III-V_ = 4.11 (95% CI = 1.62−10.38; *z* = 2.98, *p* < 0.01)	OR_bilateral_ = 4.38 (95% CI = 1.35−14.15; *z* = 2.46, *p* < 0.05)	No difference (*p* = 0.554)
Wiggling-Oscillating Movements	53 (11.3%)	45 (9.6%)	370 (79.1%)	OR_GMFCS III-V_ = 3.58 (95% CI = 1.55−8.26; *z* = 2.99, *p* < 0.01)	No difference (*p* = 0.059)	No difference (*p* = 0.821)
Kicking	55 (11.8%)	170 (36.3%)	243 (51.8%)	OR_GMFCS III-V_ = 2.60 (95% CI = 1.40−4.85; *z* = 3.01, *p* < 0.01)	No difference (*p* = 0.683)	No difference (*p* = 0.183)
Excitement Bursts	17 (3.6%)	19 (4.1%)	432 (92.3%)	OR_GMFCS III-V_ = 5.57 (95% CI = 1.30−23.93; *z*=2.31, *p* < 0.05)	OR_bilateral_ = 5.13 (95% CI = 1.23−21.36; *z* = 2.25, *p* < 0.05)	No difference (*p* = 0.455)
Smiles	94 (20.1%)	21 (4.5%)	353 (75.4)	OR_GMFCS III-V_ = 2.84 (95% CI = 1.01−7.96; *z* = 1.99, *p* < 0.05)	OR_bilateral_ = 5.38 (95% CI = 1.18−24.53; *z*=2.18, *p* < 0.05)	No difference (*p* = 0.537)
Mouth Movements	83 (17.7%)	147 (31.4%)	238 (50.9%)	OR_GMFCS III-V_ = 6.50 (95% CI = 3.44−12.32; *z* = 5.75, *p* < 0.001)	OR_bilateral_ = 4.84 (95% CI = 2.37-9.88; *z* = 4.32, *p* < 0.001)	No difference (*p* = 0.395)
Tongue Movements	n.a.	134 (28.6%)	334 (71.4%)	OR_GMFCS III-V_ = 1.65 (95% CI = 1.06−2.56; *z* = 2.22, *p* < 0.05)	No difference (*p* = 0.705)	No difference (*p* = 0.552)
Side-to-Side Movements of the Head	114 (24.4%)	105 (22.4%)	245 (53.2%)	OR_GMFCS III-V_ = 3.43 (95% CI = 1.92−6.13; *z* = 4.17, *p* < 0.001)	OR_bilateral_ = 3.12 (95% CI = 1.60−6.10; *z* = 3.33, *p* < 0.001)	No difference (*p* = 0.216)
Hand-to-Mouth Contact	65 (13.9%)	20 (4.3%)	383 (81.8%)	OR_GMFCS III-V_ = 3.73 (95% CI = 1.27−10.98; *z* = 2.39, *p* < 0.05)	No difference (*p* = 0.525)	No difference (*p* = 0.136)
Hand-to-Hand Contact	27 (5.8%)	18 (3.8%)	423 (90.4%)	No difference (*p* = 0.143)	No difference (*p* = 0.917)	No difference (*p* = 0.250)
Fiddling	40 (8.5%)	10 (2.1%)	418 (89.4%)	No difference (*p* = 0.308)	No difference (*p* = 0.204)	No difference (*p* = 0.451)
Reaching	0	n.a.	468 (100%)	n.a.	n.a.	n.a.
Foot-to-Foot Contact	38 (8.1%)	81 (17.3%)	349 (74.6%)	OR_GMFCS III-V_ = 9.37 (95% CI = 3.65−24.07; *z* = 4.65, *p* < 0.001)	OR_bilateral_ = 5.32 (95% CI = 2.25−12.55; *z*=3.81, *p* < 0.001)	No difference (*p* = 0.558)
Legs Lift	40 (8.5%)	40 (8.5%)	388 (83%)	OR_GMFCS III-V_ = 3.86 (95% CI = 1.53−9.75; *z* = 2.85, *p* < 0.01)	OR_bilateral_ = 4.67 (95% CI = 1.51−14.46; *z*=2.67, *p* < 0.01)	No difference (*p* = 0.127)
Segmental Movements of Fingers and Wrists	n.a.	64 (13.7%)	404 (86.3%)	OR_GMFCS I-II_ = 16.96 (95% CI = 8.10−35.50; *z* = 7.51, *p* < 0.001)	OR_unilateral_ = 105.20 (95% CI = 42.30−261.60; *z* = 10.02, *p* < 0.001)	No difference (*p* = 0.080)
Arching	8 (1.7%)	33 (7.1%)	427 (91.2%)	OR_GMFCS III-V_ = 26.00 (95% CI = 2.73−248.02; *z* = 2.83, *p* < 0.01)	OR_bilateral_ = 12.08 (95% CI = 2.05−71.12; *z*=2.76, *p* < 0.01)	No difference (*p* = 0.704)
Rolling to Side	5 (1%)	42 (9%)	421 (90%)	OR_GMFCS III-V_ = 9.00 (95% CI=1.23−65.64; *z* = 2.17, *p* < 0.05)	OR_bilateral_ = 13.33 (95% CI=1.36−130.92; *z* = 2.22, *p* < 0.05)	No difference (*p* = 0.663)
Visual Exploration	158 (33.8%)	97 (20.7%)	213 (45.5%)	OR_GMFCS III-V_ = 4.32 (95% CI = 2.39−7.79; *z* = 4.86, *p* < 0.001)	No difference (*p* = 0.061)	No difference (*p* = 0.112)
Hand Regard	21 (4.5%)	n.a.	447 (95.5%)	OR_GMFCS I-II_ = 4.04 (95% CI = 1.60−10.22; *z* = 2.95, *p* < 0.01)	OR_unilateral_ = 6.12 (95% CI = 2.49−15.01; *z* = 3.96, *p* < 0.001)	No difference (*p* = 0.679)
Head Anteflexion	2 (0.4%)	10 (2.1%)	456 (97.5%)	No difference (*p* = 0.061)	No difference (*p* = 0.216)	No difference (*p* = 0.612)
Circular Arm Movements	n.a.	60 (12.8%)	408 (87.2%)	OR_GMFCS III-V_ = 18.71 (95% CI = 4.51−77.68; *z* = 4.03, *p* < 0.001)	OR_bilateral_ = 16.94 (95% CI =2.31−123.93; *z* = 2.79, *p* < 0.01)	OR_non-spastic_ = 8.77 (95% CI = 4.08−18.87; *z* = 5.56, *p* < 0.001)
Almost No Leg Movements	n.a.	32 (6.8%)	436 (93.2%)	OR_GMFCS III-V_ = 5.55 (95% CI = 1.66−18.51; *z* = 2.79, *p* < 0.01)	No difference (*p* = 0.088)	No difference (*p* = 0.879)

The three last columns provide the odds ratios (OR), 95% confidence intervals (CI), *z*-statistics and *p*-values for the outcome variables activity limitation, topography, and motor type of CP; the references for ORs are subscript. Key: ^a^ Twenty individuals with mixed type CP (spasticity and dyskinesia) were excluded.

**Table 5:** Postural patterns at 3–5 months and their normal vs. atypical occurrence in 468 infants later diagnosed with cerebral palsy (CP).

Postural Pattern	Normal *n* (%)	Atypical *n* (%)	Not Observed *n* (%)	GMFCS-E&RLevels I–II (*n* = 162; 34.6%) vs. III–V (*n* = 306; 65.4%)	Unilateral (*n* = 92; 19.7%) vs. Bilateral (*n* = 376; 80.3%) CP	Spastic (*n* = 415; 92.6%) vs. Non-spastic (*n* = 33; 7.4%) CP ^a^
Head Centered	182 (38.9%)	286 (60.1%)	n.a.	OR_GMFCS III-V_ = 3.29 (95% CI = 2.21−4.89; *z*=5.87, *p* < 0.001)	OR_bilateral_ = 3.09 (95% CI = 1.93−4.94; *z* = 4.70, *p* < 0.001)	No difference (*p* = 0.261)
Body Symmetry	164 (35%)	304 (65%)	n.a.	OR_GMFCS III-V_ = 2.47 (95% CI = 1.66−3.68; *z* = 4.48, *p* < 0.001)	No difference (*p* = 0.120)	OR_non-spastic_ = 3.30 (95% CI = 1.25−8.74; *z* = 2.41, *p* < 0.05)
ATN Posture	351 (75%)	117 (25%)	n.a.	OR_GMFCS III-V_ = 2.08 (95% CI =1.29−3.37; *z* = 2.99, *p* < 0.01)	OR_bilateral_ = 1.91 (95% CI = 1.05−3.48; *z* = 2.12, *p* < 0.05)	No difference (*p* = 0.087)
Flat Posture	n.a.	67 (14.3%)	401 (85.7%)	No difference (*p* = 0.107)	No difference (*p* = 0.821)	No difference (*p* = 0.359)
Variability of Finger Postures	50 (10.7%)	418 (89.3%)	n.a.	OR_GMFCS III-V_ = 5.96 (95% CI = 3.11−11.43; *z* = 5.37, *p* < 0.001)	No difference (*p* = 0.127)	No difference (*p* = 0.138)
Predominant Fisting	n.a.	175 (37.4%)	293 (62.6%)	No difference (*p* = 0.627)	No difference (*p* = 0.084)	No difference (*p* = 0.056)
Synchronized Opening and Closing of Fingers	n.a.	41 (8.8%)	427 (91.2%)	No difference (*p* = 0.074)	No difference (*p* = 0.197)	No difference (*p* = 0.217)
Finger Spreading	n.a.	87 (18.6%)	381 (81.4%	OR_GMFCS III-V_ = 2.76 (95% CI = 1.54−4.93; *z* = 3.43, *p* < 0.001)	No difference (*p* = 0.327)	No difference (*p* = 0.499)
Hyperextension of Neck and/or Trunk	n.a.	94 (20.7%)	371 (79.3)	OR_GMFCS III-V_ = 3.29 (95% CI = 1.85−5.84; *z* = 4.05, *p* < 0.001)	OR_bilateral_ = 3.26 (95% CI = 1.52−6.98; *z* = 3.03, *p* < 0.01)	No difference (*p* = 0.847)
Extended Arms	n.a.	76 (16.1%)	392 (83.9%)	No difference (*p* = 0.158)	No difference (*p* = 0.966)	No difference (*p* = 0.103)
Extended Legs	n.a.	124 (26.5%)	344 (73.5%)	OR_GMFCS III-V_ = 2.06 (95% CI = 1.29−3.30; *z* = 3.03, *p* < 0.01)	No difference (*p* = 0.054)	No difference (*p* = 0.130)

The three last columns provide the odds ratios (OR), 95% confidence intervals (CI), *z*-statistics and *p*-values for the outcome variables activity limitation, topography, and motor type of CP; the references for ORs are subscript. Key: ^a^ Twenty individuals with mixed type CP (spasticity and dyskinesia) were excluded.

**Table 6:** The movement character assessed at 3–5 months [5] and its rate of occurrence in 468 infants later diagnosed with cerebral palsy (CP).

Movement Character	Yes *n* (%)	No *n* (%)	GMFCS-E&R Levels I–II (*n* = 162; 34.6%) vs. III–V (*n* = 306; 65.4%)	Unilateral (*n* = 92; 19.7%) vs. Bilateral (*n* = 376; 80.3%) CP	Spastic (*n* = 415; 92.6%) vs. Non-spastic (*n* = 33; 7.4%) CP ^a^
Smooth and Fluent	1 (0.3%)	467 (99.7%)	n.a.	n.a.	n.a.
Cramped-Synchronized	88 (18.8%)	380 (81.2%)	OR_GMFCS III-V_ = 11.69 (95% CI = 4.63−29.48; *z* = 5.21, *p* < 0.001)	OR_bilateral_ = 27.39 (95% CI = 3.76−199.46; *z* = 3.27, *p* < 0.01)	No difference (*p* = 0.707)
Monotonous	385 (82.3%)	83 (17.7%)	OR_GMFCS III-V_ = 1.67 (95% CI = 1.03−2.71; *z* = 2.09, *p* < 0.05)	OR_bilateral_ = 1.76 (95% CI = 1.02−3.03; *z* = 2.02, *p* < 0.05)	No difference (*p* = 0.575)
Jerky	106 (22.6%)	362 (77.4%)	No difference (*p* = 0.090)	No difference (*p* = 0.276)	No difference (*p* = 0.058)
Stiff	264 (56.4%)	204 (43.6%)	OR_GMFCS III-V_ = 2.89 (95% CI = 1.95−4.28; *z* = 5.29, *p* < 0.001)	OR_bilateral_ = 2.26 (95% CI = 1.42−3.61; *z* = 3.44, *p* < 0.001)	No difference (*p* = 0.113)
Tremulous	76 (16.2%)	392 (83.8%)	OR_GMFCS III-V_ = 2.69 (95% CI = 1.45−4.97; *z* = 3.15, *p* < 0.01)	No difference (*p* = 0.198)	No difference (*p* = 0.381)
Predominantly Slow	84 (17.9%)		No difference (*p* = 0.113)	No difference (*p* = 0.283)	No difference (*p* = 0.547)
Predominantly Fast	26 (5.6%)		No difference (*p* = 0.295)	No difference (*p* = 0.275)	No difference (*p* = 0.614)

The three last columns provide the odds ratios (OR), 95% confidence intervals (CI), *z*-statistics and *p*-values for the outcome variables activity limitation, topography, and motor type of CP; the references for ORs are subscript. Key: ^a^ Twenty individuals with mixed type CP (spasticity and dyskinesia) were excluded.
